# A Randomized Placebo Controlled Trial of Ibuprofen for Respiratory Syncytial Virus Infection in a Bovine Model

**DOI:** 10.1371/journal.pone.0152913

**Published:** 2016-04-13

**Authors:** Paul Walsh, Nicole Behrens, Francisco R. Carvallo Chaigneau, Heather McEligot, Karan Agrawal, John W. Newman, Mark Anderson, Laurel J. Gershwin

**Affiliations:** 1 Department of Emergency Medicine, Division of Pediatric Emergency Medicine, Sutter Medical Center Sacramento, Sacramento, California, United States of America; 2 Department of Pathology, Microbiology, and Immunology, School of Veterinary Medicine, University of California Davis, 1 Shields Ave, Davis, California, United States of America; 3 California Animal Health and Food Safety Laboratory, San Bernardino branch, 105 W Central Ave, San Bernardino, California, United States of America; 4 Obesity and Metabolism Research Unit, United States Department of Agriculture, Agricultural Research Service, Western Human Nutrition Research Center, Davis, California, United States of America; 5 Department of Nutrition, University of California Davis, Davis, 95616 California, United States of America; 6 NIH West Coast Metabolomics Center, University of California Davis, Davis, California, United States of America; 7 California Animal Health and Food Safety Laboratory, 620 W. Health Sciences Drive, Davis, California, United States of America; Imperial College London, UNITED KINGDOM

## Abstract

**Background:**

Respiratory syncytial virus (RSV) is the most common cause of bronchiolitis and hospital admission in infants. An analogous disease occurs in cattle and costs US agriculture a billion dollars a year. RSV causes much of its morbidity indirectly via adverse effects of the host response to the virus. RSV is accompanied by elevated prostaglandin E_2_ (PGE_2_) which is followed by neutrophil led inflammation in the lung. Ibuprofen is a prototypical non-steroidal anti-inflammatory drug that decreases PGE_2_ levels by inhibiting cyclooxygenase.

**Hypotheses:**

We hypothesized that treatment of RSV with ibuprofen would decrease PGE_2_ levels, modulate the immune response, decrease clinical illness, and decrease the histopathological lung changes in a bovine model of RSV. We further hypothesized that viral replication would be unaffected.

**Methods:**

We performed a randomized placebo controlled trial of ibuprofen in 16 outbred Holstein calves that we infected with RSV. We measured clinical scores, cyclooxygenase, lipoxygenase and endocannabinoid products in plasma and mediastinal lymph nodes and interleukin (Il)-4, Il-13, Il-17 and interferon-γ in mediastinal lymph nodes. RSV shedding was measured daily and nasal Il-6, Il-8 and Il-17 every other day. The calves were necropsied on Day 10 post inoculation and histology performed.

**Results:**

One calf in the ibuprofen group required euthanasia on Day 8 of infection for respiratory distress. Clinical scores (p<0.01) and weight gain (p = 0.08) seemed better in the ibuprofen group. Ibuprofen decreased cyclooxygenase, lipoxygenase, and cytochrome P450 products, and increased monoacylglycerols in lung lymph nodes. Ibuprofen modulated the immune response as measured by narrowed range of observed Il-13, Il-17 and IFN-γ gene expression in mediastinal lymph nodes. Lung histology was not different between groups, and viral shedding was increased in calves randomized to ibuprofen.

**Conclusions:**

Ibuprofen decreased PGE_2_, modulated the immune response, and improved clinical outcomes. However lung histopathology was not affected and viral shedding was increased.

## Introduction

Respiratory syncytial virus (RSV) is the most common cause of bronchiolitis in infants. The clinical manifestations largely reflect the hosts’ immune response to RSV; authors have described eicosanoid and cytokine storms leading to neutrophilic infiltration and tissue destruction. Antibodies formed to bystander antigens lead to bronchospasm on subsequent re-exposure.[1] Eicosanoid pathway products in general, and prostaglandin E_2_ (PGE_2_) in particular are higher in more severe RSV infection in infants.[2] PGE_2_ is produced by cyclooxygenase (COX)-2 in respiratory epithelial cells in response to RSV infection.[3] Multiple COX- and lipoxygenase (LOX)-generated mediators stimulate the cytokine responses affecting neutrophil and monocyte trafficking as well as stimulation of mucus secretion from bronchial goblet cells.[4–6]

COX-1 is constitutive, but COX-2 is mostly inducible in the lung;[7] and COX-2 in particular is believed responsible for the production of prostaglandins and thromboxanes in RSV.[8] Simple nonsteroidal anti-inflammatory drugs (NSAIDs) such as ibuprofen inhibit COX-2 and to a lesser extent COX-1. Ibuprofen is inexpensive and widely available for human use. Current treatment of human RSV bronchiolitis is mostly symptomatic and supportive with modest benefit from bronchodilators in some patients, but not from glucocorticoids or leukotriene inhibitors.[9–12] NSAIDs are not currently used in human RSV and aspirin is contraindicated in infants.[13, 14] However pretreatment of RSV infected cotton rats with indomethacin decreases the severity of lung histology,[15] and in veterinary medicine NSAIDs are used as adjuncts to antibiotics in mixed respiratory infections in cattle and piglets.[16–18] A randomized controlled trial (RCT) in asthmatic children found that ibuprofen, compared with acetaminophen, decreased near term wheezing following viral upper respiratory tract infection (URI)[19] and ibuprofen decreases antibody production in human monocytes.[20]

The outbred Holstein bovine model of RSV bronchiolitis uses a naturally occurring bovine RSV to replicate the clinical, immunological, and histological features of human RSV. This model has good applicability to human infants and has replicated the enhanced disease observed in formalin killed RSV vaccine.[21]

We hypothesized that treatment of RSV with ibuprofen would decrease systemic PGE_2_ levels, modulate the immune response, decrease clinical illness, and decrease the histopathological lung changes in a bovine model of RSV. We further hypothesized that viral replication would be unaffected. These hypotheses were tested in a placebo controlled RCT.

## Materials and Methods

We performed an RCT using a bovine model to test the hypothesis that ibuprofen would decrease the severity of RSV bronchiolitis. The University of California Davis Institutional Animal Care and Use Committee approved this study (Protocol #17771).

### Subjects

Sixteen five to six week-old outbred pre-ruminant bottle-fed Holstein bull calves.

### Intervention

Following a week of observation to ensure they were disease free, calves were experimentally infected with 5 mL of 1.5 X 10^5/ml (by TCID50) bovine RSV using a tightly fitting mask with an attached nebulizer.[21, 22] The calves were randomized to receive either ibuprofen 10mg/kg three times daily or identical appearing and tasting placebo administered in milk replacer. This dose is the same as is typically used in children (10 mg/kg tid) and is similar dosing that has previously been used in cattle. Randomization was performed using a minimization strategy based first on maternal RSV antibody titres (determined by indirect immunofluorescence) and then body weight. All animals were housed in climate controlled barns at our veterinary school. Free access to water and a small amount of oat hay was allowed.

### Measurement of clinical scores

A complete physical exam was performed at 07:00 daily, with additional respiratory exams and temperature measurements at 13:00 and 19:00. These findings were used to calculate a daily clinical severity of illness score. This score was originally developed by Collie et al [23] and has been widely used subsequently. A modified version which underweights the importance of fever has also been described,[22] and because of the antipyretic effects of ibuprofen, that is the version we used. The scoring schema is in supporting information (S1 Table). The veterinarian and physician doing evaluations and their research assistants were blinded to drug allocation. The calves were weighed at the start and the end of the experiment. On day 10 post infection the calves were euthanized with pentobarbital overdose and necropsied.

### Measurement of the eicosanoid, thromboxane, lipoxygenase and endocannabinoid pathway products

Lipid mediators of immune function were quantified using liquid chromatography with tandem mass spectrometry (LC-MS/MS) in plasma collected on day 0, 3, 7, and 10 of infection, and terminally collected lymph tissue. Measured mediators included oxylipins derived from cyclooxygenase, lipoxygenase, and cytochrome P450 dependent metabolism, as well as the endocannabinoid and endocannabinoid-like monoacylglycerols and acylethanolamides. Plasma (100μL) and lymph tissue (~20–30mg) were enriched with a suite of deuterated surrogates prior to extraction.[24] Plasma samples were extracted directly with 300μL acetonitrile with 1% formic acid while tissues were first cryopulverized with 50μL of methanol in a Geno/Grinder 2000 (SPEX SamplePrep; Metuchen, NJ) at 1350 rpm for 90 seconds in 30 second increments, and extracted by further pulverization with 550μL acetonitrile with 1% formic acid, and 100μL water for 90 seconds. Supernatants were isolated by centrifugation. Target analytes were isolated in extract eluents from an Ostro Sample Preparation Plate (Waters Corp; Milford, MA), concentrated into 2μL of glycerol, and reconstituted in an internal standard solution containing 1-cyclohexyl-3-ureido dodecanoic acid and 1-phenyl,3-ureido hexanoic acid in 1:1 (v/v) methanol:acetonitrile prior to analysis.[24]

Analytical targets were separated on a 2.1 x 150 mm, 1.7 μm BEH C18 column (Waters; Milford, MA) and detected by positive or negative mode electrospray ionization (endocannabinoids or oxylipins, respectively) and tandem mass spectrometry on an API 4000QTrap (Sciex; Framingham, MA).[24] Calibrants and internal standards were from Cayman Chemical (Ann Arbor, MI), or Avanti Polar Lipids Inc. (Alabaster, AL). Larodan Fine Lipids (Malmo, Sweden) provided the linoleate-derived triols 9,12,13-TriHOME and 9,10,13- TriHOME. Data was processed utilizing AB Sciex MultiQuant version 3.0.2.

### Measurement immunological profiles

Expression of interferon-γ (IFN-γ), interleukin (Il)-4, Il-17, and Il-13 was measured by real-time TaqMan RT-PCR in mediastinal lymph nodes. Expression of Il-6, Il-8 and Il-17 was also evaluated in nasal secretions as this type of sample will be readily accessible in human translation. For each gene, two primers and an internal, fluorescent labeled Roche Universal Probe Library probe was designed using Primer Express software (Applied Biosystems, Foster City, CA). Cell pellet samples were placed in 470μL of Buffer RLT+Isopropanol and 40uL Proteinase K Enzyme, heated at 56°C for 30 minutes, and stored at -20°C. Two grinding beads (4 mm diameter, stainless steel beads, SpexCertiprep, Metuchen, NJ) were added and the tissues homogenized in a GenoGrinder2000 (SpexCertiprep) for 2.5 minutes at 1000 strokes per minute. Total RNA was extracted from the tissue lysates using a BioSprint automated nucleic acid (ANA) workstation (Qiagen) according to the manufacturer’s instructions for the One-for-all Vet Kit (Qiagen).

The Quantitect Reverse transcription kit (Qiagen) was used for cDNA synthesis following the manufactures directions with the following modifications. Ten microliters of RNA were digested with 1μL of gDNA WipeOut Buffer by incubation at 42°C for five minutes and then briefly centrifuged. Genomic DNA contamination was tested by using 1μL of digested RNA and running the real-time PCR housekeeping gene. Then 0.5 μL of Quantitect Reverse Transcriptase, 2μL Quantitect RT buffer, 0.5μL RT Primer Mix, 0.5μL 20 pmol Random Primers (Invitrogen) were added and brought up to a final volume of 20μL and incubated at 42°C for 40 minutes. The samples were inactivated at 95°C for 3 minutes, chilled, and 80μL of water was added.

Final quantitation was done using the comparative CT method (User Bulletin #2, Applied Biosystems) and is reported as relative transcription or the n-fold difference relative to a calibrator cDNA (i.e. lowest target gene transcription). In brief, the three reference genes were averaged to normalize the CT values of the target genes (ΔCT). The ΔCT was calibrated against the average of the negative control group within each target gene. The linear amount of target molecules relative to the calibrator was calculated by 2^-ΔΔCt^ and gene transcription is expressed as an n-fold difference relative to the calibrator.

### Measurement of histopathology

Lung consolidation was estimated by inspection by an experienced board certified veterinary pathologist. Sections of the right lung were collected and fixed for at least 24 hours in 10% formalin. The left lung was then perfused with formalin at 15 cm water pressure. A second board certified veterinary pathologist blinded to the gross autopsy findings performed detailed histological assessment of the slides. Both pathologists were blinded to drug assignment. A scoring system (S2 Table) was derived from the placebo group and validated on weight change and clinical findings. This scoring system was then applied to both groups. The scoring system is described in detail elsewhere. Briefly it comprises three components: a semi-quantitative estimate of percentage lung consolidation; a semi-quantitative estimate of acute inflammatory processes as evidenced by neutrophilic infiltrates, transmigration of neutrophils into the bronchial and bronchiolar lumen, and septal thickening with edema and cellular exudate; and a semi-quantitative estimate of sub-acute lung inflammation as evidence by mononuclear cell infiltrates. The histopathological scoring system is described in detail in the supporting information files. We also compared the associations between each of these components by treatment group.

### Measurement of viral shedding

We took nasal swabs daily and quantitated viral shedding with RT-PCR. Total RNA was extracted from the cell lysate from calf nasal swabs collected and stored in lysis buffer (Life Technologies, NY), using the Pure Link RNA min kit (Life Technologies, NY), according the manufactures directions.

Extracted RNA was stored at -80°C until use. Viral cDNA was synthesized by using SuperScript III First Strand synthesis system (Invitrogen, CA), according to the manufacturer’s directions. The cDNA thermocycling program consisted of 10 minutes at 25°C, 50 minutes at 50°C, and a termination cycle of 85°C of 5 minutes. Synthesized cDNA was stored at -20°C. The Q-RT-PCR was performed in triplicate on a 384-well plate, in a 20uL reaction volume. The 20uL reaction mixture contained 10uL Sybr green qPCR master mix, 2uL of BRSV-NP-F primer, (GCAATGCTGCAGGACTAGGTATAAT) 2uL BRSV-NP-R reverse primer (ACACTGTAATTGATGACCCCATTC), and 2uL nuclease free water. The RT-PCR thermocycling program consisted of 48°C for 30 min, 95°C for 5 minutes, followed by 40 cycles of 95°C and 55°C for 1 minute each. Fluorescence was measured following each cycle and displayed graphically (AB Applied Biosystems ViiA-7 detection software, version 1.1) The ViiA-7 software determined a cycle threshold (Ct) value, which identifies the first cycle at which the fluorescence is detected above the baseline for each sample or standard.

### Statistical Methods

Data entry was performed using a customized Filemaker-pro 12 database (Filemaker Inc. Santa Clara, CA). Serial data was analyzed using mixed effects models. Non-normally distributed variables were compared using rank sum techniques. Categorical variable were compared using Fisher’s exact test. These tests were performed with Stata 14 (Statacorp LLP, College Station, TX). All clinical, immunological, and metabolomics data were then included in a partial-least squares discriminant analysis (PLS-DA), using treatment as the classifier. PLS-DA is a classification tool that is widely used when there are more independent variables than study subjects. This situation commonly arises in studies of lipid mediators and in genetic studies. The multivariate analyses and associated normality transformations were performed using imDEV v1.42, a Microsoft Excel (Microsoft Corporation, Redmond, WA) Add-In interface [25] to the R-statistical environment (R Foundation for Statistical Computing, Vienna, Austria).

## Results and Discussion

**Fig 1:**
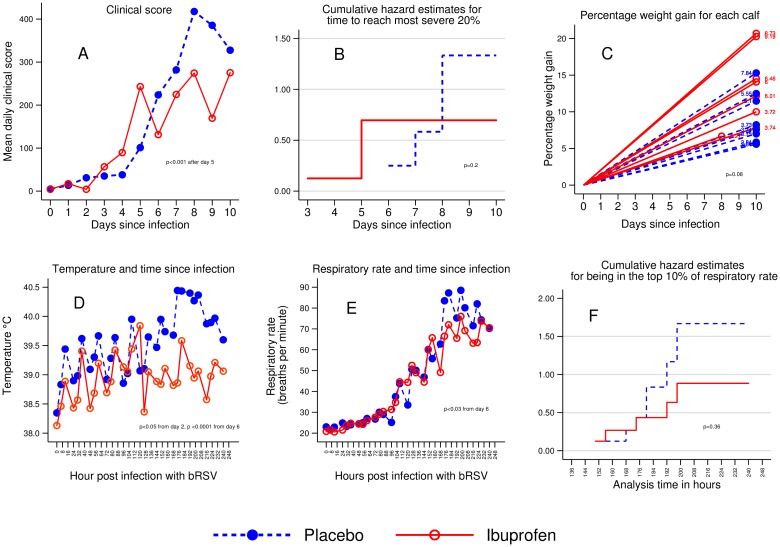
Comparison of clinical outcomes in ibuprofen and placebo groups. Sixteen calves were experimentally infected with bovine respiratory syncytial virus by nebulizer on Day 0. Eight received ibuprofen and received eight placebo. (A) Shows the mean clinical score by treatment group for each day. The groups were statistically significantly different by Day 5 using a mixed effects model. (B) Shows the time taken to enter the most severe decile of illness as measured by the clinical score. Statistical significance was calculated using the log rank test. (C) Shows the percentage weight gain for each individual calf. Calves randomized to ibuprofen are in red; placebo is in blue. Statistical significance was calculated using ordinary least squares regression. (D) Shows the mean temperature by treatment group. Temperature was measured every eight hours. (E) Shows the mean respiratory rate per group. Respiratory rate was measured every eight hours. Statistical significance was calculated for both temperature and respiratory rate using a mixed effect model. (F) Shows the time taken to enter the most severe decile of tachypnea. Statistical significance for (F) was calculated using the log rank test.

Sixteen calves were randomized; 15 completed the protocol as scheduled on day 10. One, in the ibuprofen group, was euthanized for respiratory distress on day 8. The mean and peak clinical scores were lower (*p* <0.001) and the mean weight gain 4 kg higher in the ibuprofen than the placebo group (*p* = 0.08 Decreased pyrexia and lower respiratory rates in the ibuprofen group had the greatest influence on clinical scores. The ibuprofen group had noticeably less sick behaviour, (depressed, failure to rise, anorexia) than the placebo group. The clinical scores and time to more severe clinical scores are shown in Fig 1.

**Fig 2:**
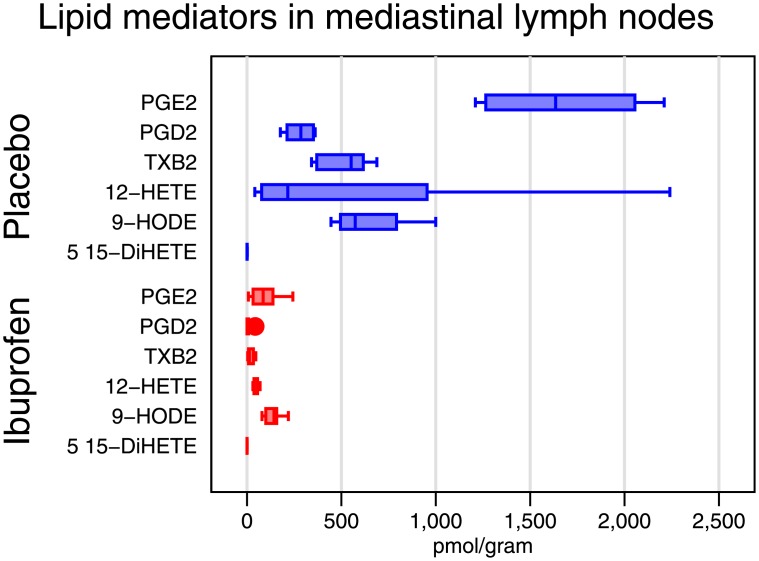
Comparison of mediastinal lymph node lipid mediators as measured by mass spectroscopy for each treatment group. PGE_2_; Prostaglandin E_2,_ TXB; Thromboxane B_2_, PGD_2;_ Prostaglandin D_2,_ 12-HETE; 12-Hydroxyeicosatetraenoic acid, 9-HODE; 9-Hydroxy-10,12-octadecadienoic acid (9-HODE), 5,15-DiHETE; 5,15-dihydroxyeicosatetraenoic acid. Ibuprofen is in red, placebo in blue.

Mediastinal lymph node levels of cyclooxygenase-derived products were markedly decreased in the ibuprofen group, with PGE_2_ >>Thromboxane (TX) B2 > PGD_2_ > 9-Hydroxy-10,12-octadecadienoic acid (9-HODE) >>>5,15-dihydroxyeicosatetraenoic acid (5,15-DiHETE). Levels of the 12-lipoxygenase product, 12-Hydroxyeicosatetraenoic acid (12-HETE) were highly variable, but also showed substantial reductions in the ibuprofen group. Thromboxane A2 and the lipoxygenase pathways were also markedly inhibited in the ibuprofen group. Fig 2 shows the effect on selected individual components of each pathway. The complete set of measured mediators is in S3 and S4 Tables.

**Fig 3:**
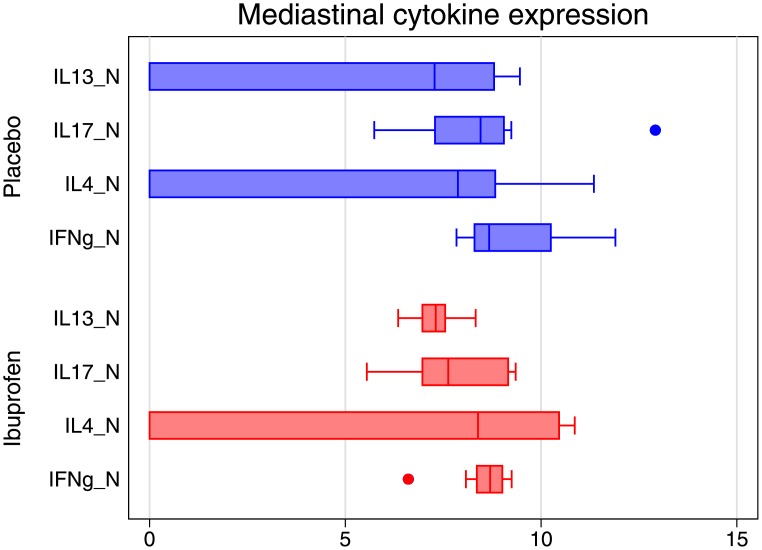
Comparison of mean cytokine expression as measured by polymerase chain reaction of the genes required for their production in mediastinal lymph nodes for each treatment group. Ibuprofen is in red, placebo in blue.

**Fig 4:**
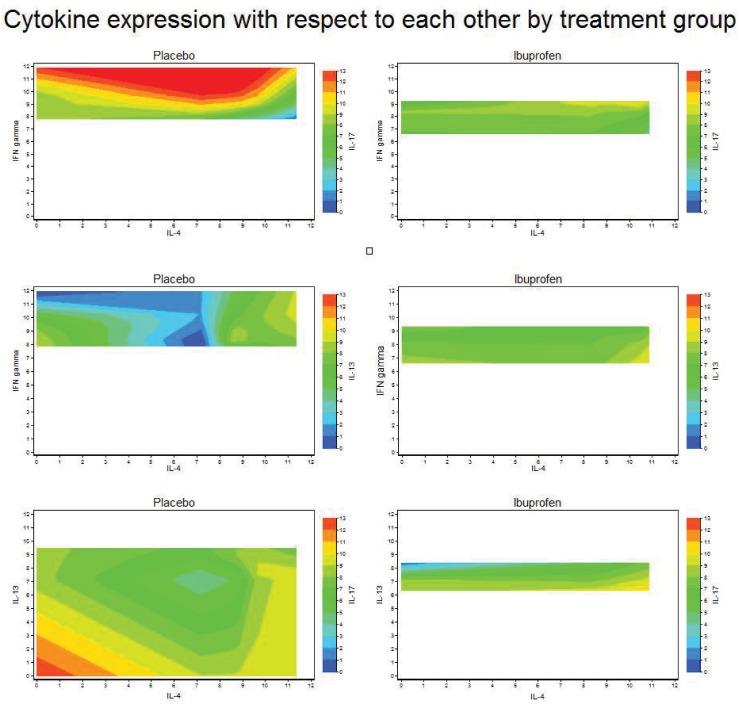
Comparison of the expression of each cytokine (as measured by polymerase chain reaction of the genes required for their production) with respect to the expression of other cytokines in mediastinal lymph node in ibuprofen and placebo groups. Warmer colors (yellows, reds) on the Z-axis indicate higher levels, cooler color (greens, blues) indicate lower levels.

Ibuprofen’s effect on the cytokine profiles of the bronchial lymph nodes was complex. Rather than seeing straightforward changes in Th-1/Th-2 skew we observed a generalized moderation of cytokine expression across IFN-γ, Il-17 and Il-13 functions with respect to each other and Il-4. Figs 3 and 4 show cytokine expression in the mediastinal lymph nodes of animals treated with ibuprofen tended towards a narrow range compared with the placebo group.

**Fig 5:**
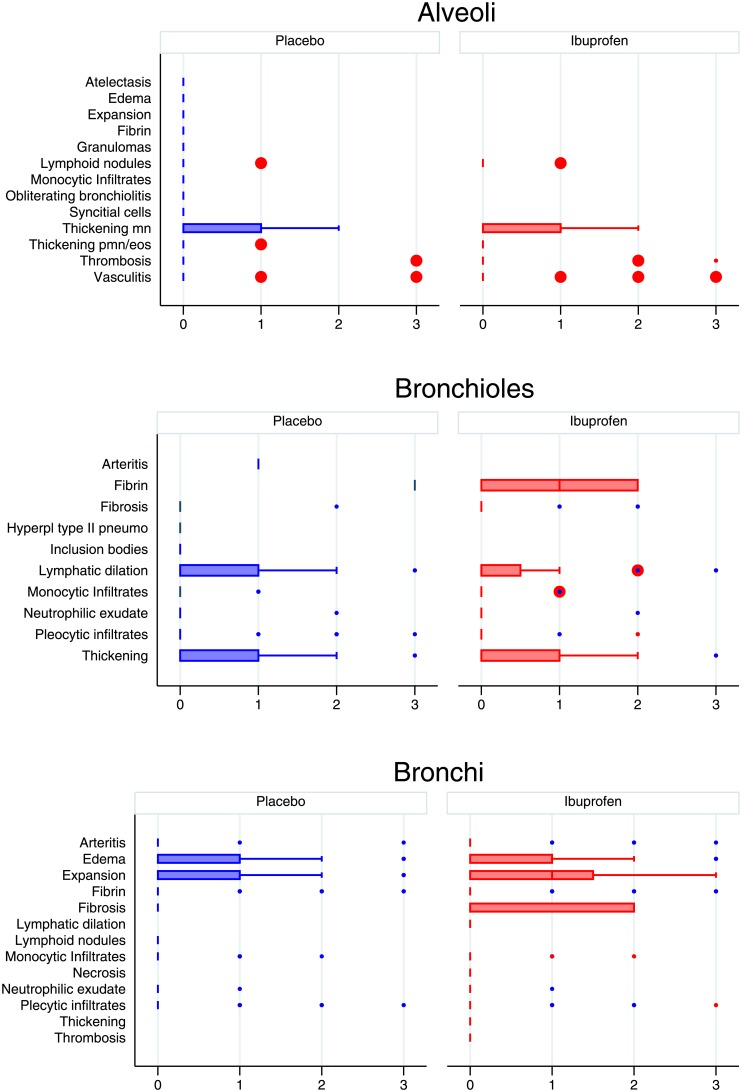
Comparison of histology findings between placebo and ibuprofen treated calves broken down by anatomical levels as follows: alveoli, bronchioles, and bronchi. These are scored 0 (absent), 1 (mild), 2 (moderate) 3 (severe). The median is indicated by a line and the interquartile range by the box. Whiskers encompass data 1.5 times the IQR of the upper and lower quartiles. Outlying data is indicated with points. Ibuprofen is in red, placebo in blue. EOS; eosinophils, Hyperpl; hyperplastic, MN; monocytes, PMN; neutrophils, Pneumo; pneumocytes.

**Fig 6:**
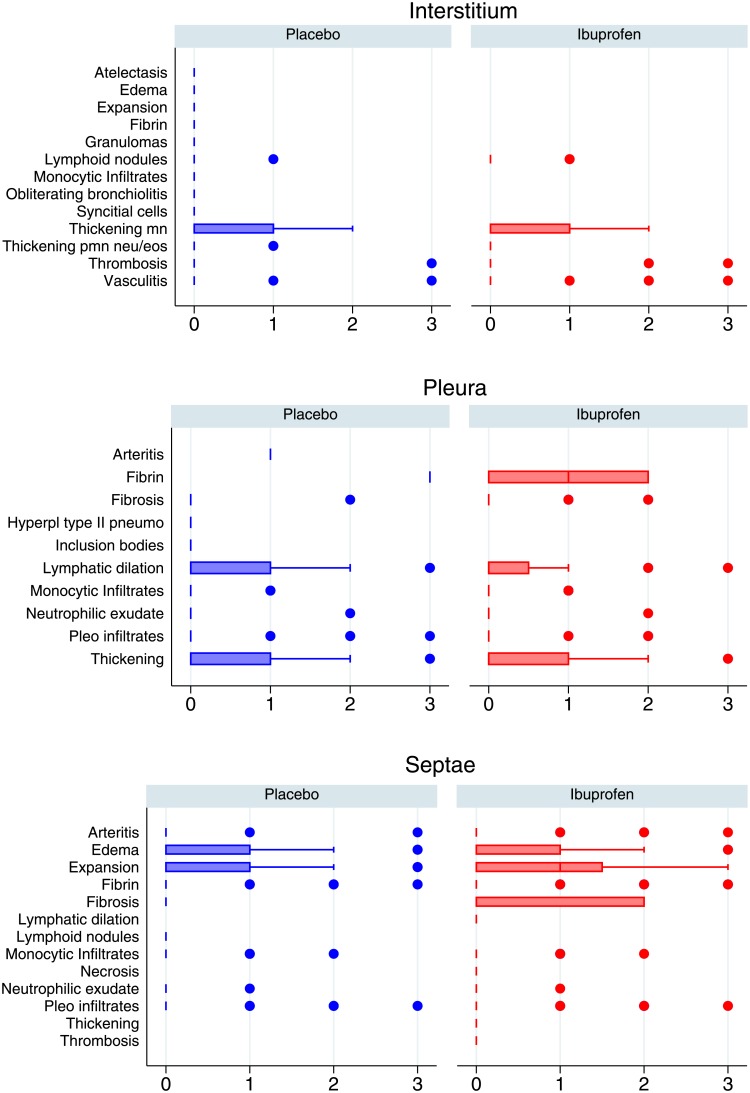
Comparison of histology findings between placebo and ibuprofen treated calves broken down by anatomical levels as follows: interstitium, septae, and pleura. These are scored 0 (absent), 1 (mild), 2 (moderate) 3 (severe). The median is indicated by a line and the interquartile range by the box. Whiskers encompass data 1.5 times the IQR of the upper and lower quartiles. Outlying data is indicated with points. Ibuprofen is in red, placebo in blue. EOS; eosinophils, Hyperpl; hyperplastic, MN; monocytes, PMN; neutrophils, Pneumo; pneumocytes.

**Fig 7:**
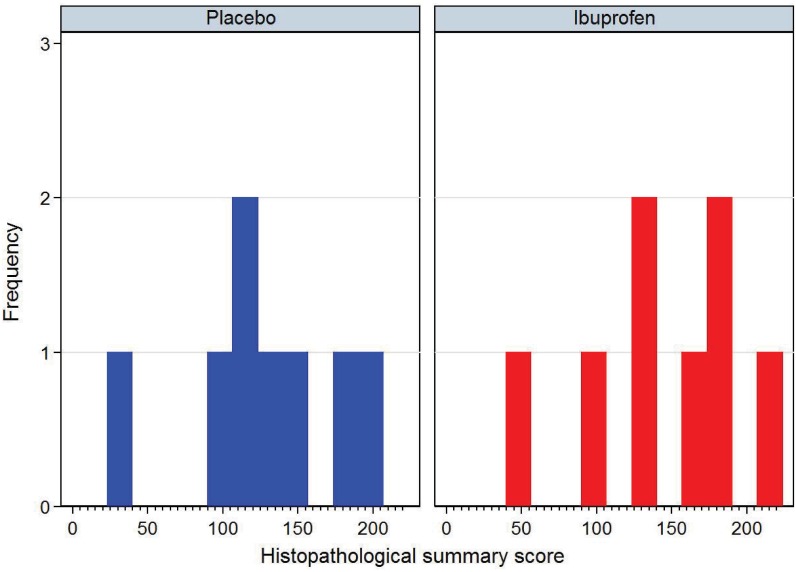
Comparison of the summary histopathological scores between placebo and ibuprofen treated calves. This summary score includes selected histological features and amount of lung consolidation. This minimum possible score is 0, the maximum possible is 632. Ibuprofen treated group is in red, placebo in blue.

There was little difference in the gross pathology or histology of the lungs between ibuprofen and placebo groups. The gross histology findings are shown in Figs 5 and 6 and overall histological scores are shown in Fig 7.

**Fig 8:**
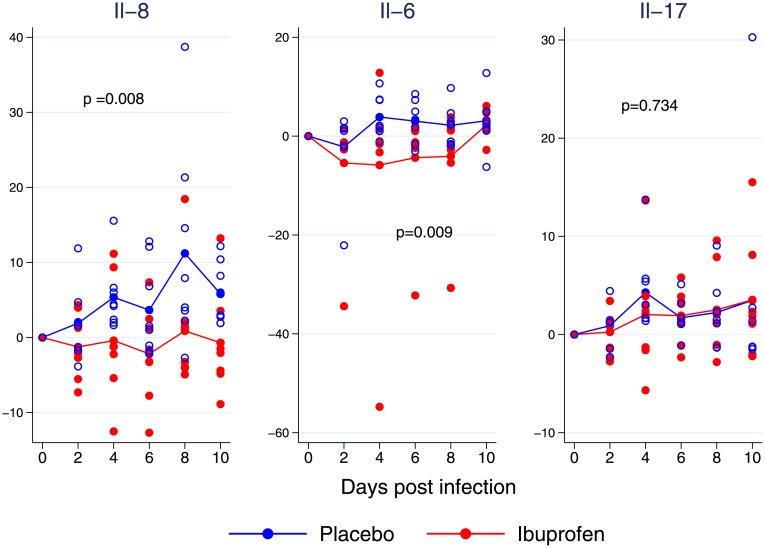
Mean by treatment group cytokine expression (samples collected by nasal swabbing). Statistical significance was calculated using a mixed effects mode. Ibuprofen is red, placebo group blue. IL-8 was significantly different between groups. IL-6 demonstrated substantial variance and return to similar levels between treatment groups over the duration of the experiment. This difference was statistically significant only at the time of peak difference (p = 0.009). Il-17 was not different between groups at any time during the experiment.

Viral shedding started on Day 3 in both groups, corresponding to the URI phase in calves and infants. Viral shedding was increased in the ibuprofen group. This difference became statistically significant (p<0.001) around the period of peak viral shedding (Fig 8).

**Fig 9:**
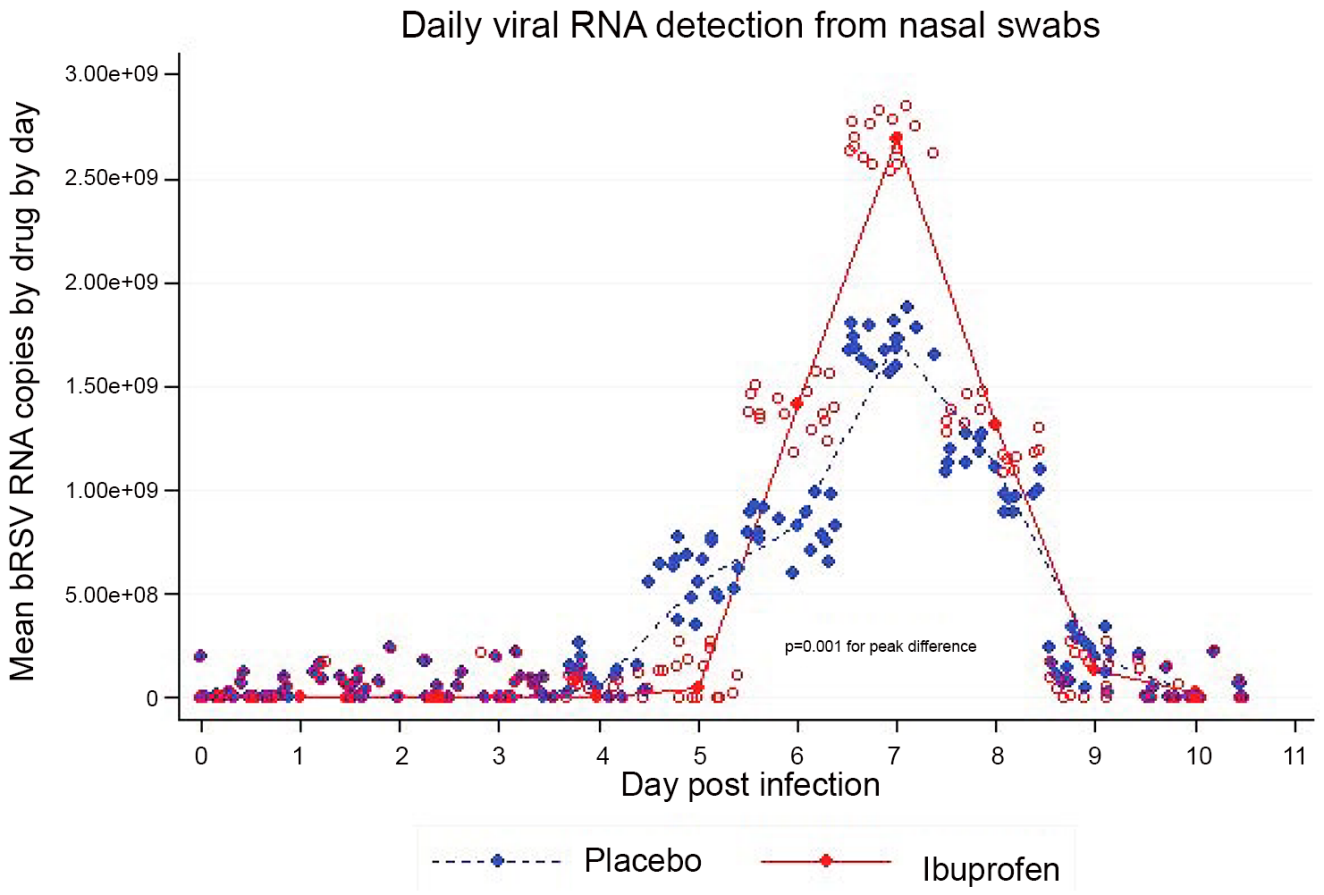
The mean by treatment group daily viral shedding as measured by polymerase chain reaction of bovine respiratory syncytial virus from nasal swabs. Each sample was tested in duplicate and both duplicates shown with random jitter added to prevent overlapping of individual measurements. Ibuprofen is red, placebo blue. Statistical significance was calculated using a mixed effects model. The difference was statistically significant on post inoculation Days 6 through 8.

This difference in viral shedding was accompanied by differences in Il-8 (*p* = 0.008). The Il-6 levels showed greater variance and were statistically significantly different only during peak illness (*p* = 0.009) as shown in Fig 9. Nasal swab Il-17 was not different between groups at any time during the experiment.

**Fig 10:**
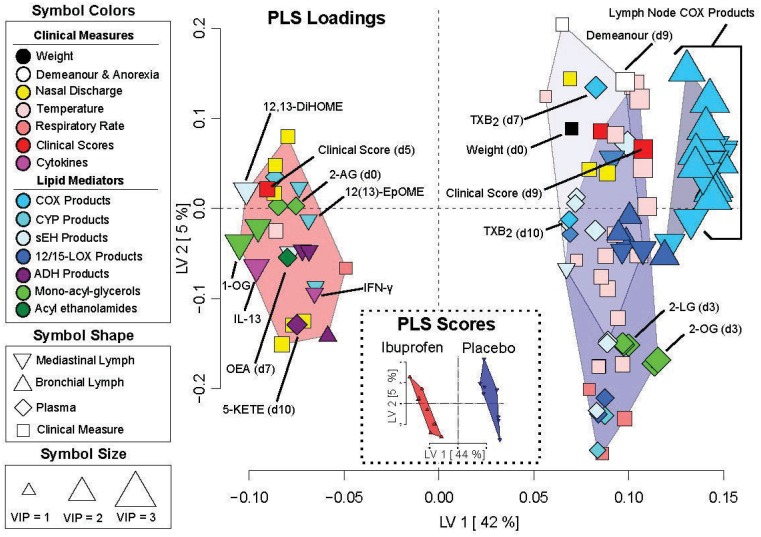
Partial least squares discriminant analysis (PLS-DA) of measurement from RSV infected calves with and without ibuprofen treatment. The Scores Plot (Inset) shows treatment group discrimination (ibuprofen: red; placebo: blue). The Loadings Plot shows the discriminant variable weighting with variables grouped by hierarchical cluster analysis of their Spearman’s correlations. Point shapes describe variable origin (i.e. tissue type or clinical observation), point colors describe variable class (i.e. metabolite or clinical data type) as described in the figure, and point sizes are defined by the variable importance in projection (VIP) scores. VIP scores roughly correlate with t-test p-values (small p = 0.12–0.05, medium p<0.05, large p<0.001). Variables to the right of the origin are elevated in the placebo group. For example, the placebo group shows higher lymph (triangles) and plasma (diamonds) concentrations of cyclooxygenase (COX) and lipoxygenase (LOX) products, higher plasma thromboxane B2 (TXB2), higher temperatures and plasma concentrations of soluble epoxide hydrolase (sEH) generated fatty acid diols at the majority of time points, but lower concentrations of fatty acid alcohol dehydrogenase (ADH) metabolites in lymph tissues and plasma.

PLS-DA analysis of metabolomics, cytokine, clinical and histological outcomes discriminated the treatment groups as seen in Fig 10. Ibuprofen lowered lymph tissue COX and LOX products, the 1- and 2- oleoylglycerol (OG), clinical scores and pyretic measures, while increasing fatty acid alcohol dehydrogenase products (i.e. fatty acid ketones), and soluble epoxide hydrolase-derived fatty acid diols (e.g. linoleate derived 12,13-dihydroxyoctadecamonoeneoic acid; 12,13-DiHOME), and the cytokine Il-13. Plasma concentrations of TXB2 were also reduced at day 7 and 10, as were numerous fatty acid diols. On day 3, plasma concentrations of 1- and 2-linoleoylglycerol (LG) and the 1- and 2-oleoylglycerol (OG) were significantly reduced by ibuprofen treatment. The full mass spectroscopy results are shown for lymph nodes in S3 Table and for plasma in S4 Table.

## Discussion

Ibuprofen decreased PGE_2_ levels and the range of individual expression of Il-13, Il-17, and IFN-γ was narrowed. This immunomodulatory effect for ibuprofen was associated with improved clinical but not histological outcomes in a bovine model of RSV bronchiolitis. Paradoxically, we also found increased RSV shedding and nasal epithelial Il-8 in the ibuprofen group.

### Lipid mediators

The rapid release of lipid mediators is an important immunological signaling process that may be dependent on constitutive COX-1 and COX-2 prior to the further induction of COX-2.[26, 27] The S-enantiomer of ibuprofen inhibits both COX-1 and COX-2 responsible for the production of a host of eicosanoids, while the R-enantiomer inhibits fatty acid amide hydrolase, which degrades acyl-ethanolamides, some of which have immunomodulatory activity.[28–33] Ibuprofen nearly eliminated COX-dependent products in lymph tissues 10 days post infection, and consistently lowered plasma thromboxane B2 levels late in the infection.

Endocannabinoids are also implicated as immunomodulatory compounds [34]. However, the measured endocannabinoids and endocannabinoid-like compounds were minimally affected in this study. While significant changes in acylethanolamides were not observed in plasma or lymph tissues, on day 7, plasma concentrations oleoyl- linoleoyl- and arachidonoyl- ethanolamides (i.e. OEA, LEA, AEA) approached a significant change (1-tailed t-test, p< = 0.1) with a calculated effect size of 0.68–0.78 (95%ci ~ -0.1–2.3), and the OEA and LEA aided in group discrimination.

Lipoxygenases also have important immunomodulatory functions during tissue injury and repair.[35] Interestingly, 12/15-LOX products were decreased in ibuprofen treated animals, while no evidence of 5-LOX activation was observed. These findings argues against an increase in substrate availability due to COX inhibition driving LOX metabolism,[36] and downstream leukotrienes[37], and a potential enhancing effect of ibuprofen on PMN 5-LOX.[38] This may be because of inter-pathway cross talk; the LOX pathways’ enzymes may be positively influenced by PGE_2_.[37, 39] We also observed a decrease in cytochrome P450 epoxidase products and an increase in their sEH-dependent diol products in the ibuprofen group. These findings mirror those of the effect of ibuprofen in human volunteer studies.[40] Our finding are also in line with those of *Palumbo et al* that noted virus dependent activation of COX and LOX12/15, and virus inhibition of 14,15-dihydroxyeicosatrienoate (14,15-DiHETrE), an sEH dependent diol, in cowpox (but not parainfluenza or herpes simplex) infection.[41] The lack of 5-LOX activation is consistent with the clinical trial findings that montelukast, which blocks the action of leukotriene D_4_ (a downstream product of 5-LOX), does not improve outcomes in bronchiolitis.

Our data suggest that ibuprofen suppresses this virus dependent activation of lipid mediators. This in turn leads to a decreased inflammatory response, which simultaneously increases viral shedding but decreases clinical illness. We speculate that this provides a biological explanation for why NSAID and aspirin use may decrease subsequent allergen induced wheezing illness.[19] Aspirin directly decreases COX and indirectly decreases LOX and the prohibition of its use in infants has been accompanied by an increase in childhood asthma.[42] There is circumstantial evidence to support this concept. Ibuprofen decreases antibody production in human cells[20] and RSV infection increases host B-cell and antibody production to bystander antigens.[43]

PGE_2_ in nanomolar concentrations can induce Th-17 differentiation through EP_2_ and EP_4_ receptors.[44] We also observed a large decrease in 12-HETE in the ibuprofen group; 12-HETE affects neutrophil calcium levels and possibly their migratory response.[45] The picture is complex because lipid mediators regulate cytokine production and vice versa as leukocytes infiltrate tissue.[46–48, 49]

### Cytokines

Experimental evidence supports a conceptual framework in which the Th-2 response to RSV, which RSV itself accentuates, increases the lung pathology of the initial infection and promotes the formation of antibodies to bystander antigens and recurrent wheeze.[1, 50] We had anticipated that by inhibiting COX-2 with ibuprofen we would drive this skew towards Th-1 with most effect on Il-4/IFN-γ ratios. In fact we observed relatively a narrower range of IFN-γ but not Il-4 gene expression in the ibuprofen group. Similarly, Il-13 and Il-17 responses to RSV infection fell within a much narrower range in the ibuprofen group. Il-17 reflects Th-17 activity. Th-17 is primarily responsible for an increased neutrophilic response and indirectly increases mucous producing goblet cells via Il-13. Therefore it may be that a goal of treatment should be to ensure adequate but not excessive Th-17 response to RSV infection.[6] We speculate that this may also be the case for Il-13, where some mucous increases respiratory epithelium resilience against infection but excessive amounts lead to mucous plugging and increased work of breathing.

### Vial shedding and nasal epithelial response

In the nasal secretions we observed lower nasal epithelial expression of Il-8 in the ibuprofen than the placebo group despite higher viral load. Increased levels of Il-8 have been associated with higher levels of viral replication.[51] Higher levels of Il-8 have also been associated with increased severity of disease as has higher viral load.[52] However PGE_2_ increases Il-8 in pulmonary microvascular endothelium.[53] These findings are consistent with human studies that have also shown an increase in RSV shedding with glucocorticoid treatment.[54, 55] Ibuprofen simultaneously increased viral load but decreased illness severity as evidenced by weight gain and clinical scores. This apparent paradox argues for a combined antiviral and immunomodulatory approach to RSV.

The balance between direct cytopathology and immune mediated lung damage may differ between species and individuals. In mice for instance lung damage is primarily immune mediated whereas in cotton rats, cattle, and humans both the virus and host response play a role.[56, 15, 57] COX inhibition also prolongs rhinovirus shedding in adult humans,[58] and decreases antibody response when given prior to vaccination.[59]

This data makes the case for an RSV treatment strategy that modestly modulates the immune response (for example with ibuprofen), while simultaneously giving drugs to prevent RSV replication. This suggestion is supported by a case report of a 4-month-old -infant with T cell-natural killer cell-B cell+ severe combined immunodeficiency and persistent RSV infection. This infant simultaneously demonstrated both a marked decrease in viral shedding and increase in pulmonary symptoms after immune system reconstitution following bone marrow transplantation; the viral shedding returned and pulmonary findings improved with immunosuppression.[56] Future research should focus on combining antiviral and NSAIDs or other immunomodulators and determining how late into the course of RSV infection treatment can be successfully started.

### Limitations

The primary limitation of our work from an agricultural stand point is that we started treatment the day of inoculation rather than waiting for clinical symptoms to develop. We did this because if ibuprofen failed to improve outcomes when given this early then our hypothesis would have been thoroughly disproven. No further work to determine how late into the course of illness NSAIDs could be beneficial would be needed. This approach mirrors that taken with other early stage drug studies. An inherent limitation of the bovine RSV model is the frequent development of secondary bacterial infections in RSV infected calves. This can be diminished by pre-treating calves with antibiotics prior to initiation of the viral infection. Secondary bacterial infection occurs less frequently in humans ranging from 20% overall to 44% in those admitted to the pediatric intensive care unit.[60–62] Consequently our model approximates the more severe forms of bronchiolitis in infants. This in part reflects our use of a minimally passaged virus that had demonstrated significant pathogenicity in the wild. Even this limitation is appropriate; most cases of RSV in both humans and cattle are mild and do not result in a clinical diagnosis of bronchiolitis. Furthermore pretreatment with antibiotics would decrease the applicability of our results to veterinary practice. Another limitation is that because we minimized on maternal anti-RSV antibody titre first and weight second our placebo group was on average heavier than the placebo group at enrollment. This tends to bias our study against finding a benefit where one exists. Our sample size was small compared to human RCTs although typical for large animal studies which are expensive to conduct. Inhibition of COX was poorly reflected in the plasma, as were systemic changes in lipoxygenase metabolites, demonstrating the difficulty in extrapolating mediator responses in the plasma to changes at the tissue level. Therefore, little information regarding the time course of lipid mediator changes can be gleaned from this study. This highlights an inherent limitation for human studies where only nasal secretions and blood can be obtained for testing, but where lung and lymph tissue are matter more. Our lipidomics findings in plasma however mirror those of human studies which have examined the effect of ibuprofen in blinded human RCTs.[40] The immunological validity of the bovine model for RSV has been demonstrated.[21]

### Implications for veterinary and medical practice

These findings generally support the early use of NSAIDS as adjunctive treatment of bovine RSV which is a key component in bovine respiratory disease complex. The results are not directly applicable to veterinary practice because our study was designed as a proof of concept with early drug administration and use of ibuprofen rather than a more widely used veterinary NSAID. Ibuprofen is short acting and has limited safety data in bovines. [63] The proof of concept design also limits its applicability to pediatrics, but ibuprofen is a widely used NSAID in pediatrics at the dosage we used. The safety of ibuprofen is well established in children. Our work does raise the caution that although NSAIDs improve clinical appearance, NSAIDs increase viral shedding with attendant implications for infection control in dairies and hospitals.

### Conclusion

Ibuprofen decreased COX, 12/15-LOX, and cytochrome P450 epoxygenase products in lung lymph nodes. Ibuprofen modulated the immune response as measured by narrowed range of observed Il-13, Il-17 and IFN-γ gene expression in mediastinal lymph nodes. Weight and clinical scores were improved by ibuprofen but lung histology was not, and viral shedding was increased. This apparent paradox argues for a combined antiviral and immunomodulatory approach to RSV.

## Supporting Information

S1 File Data files. (DTA)

S2 File Data files. (DTA)

S3 File Data files. (DTA)

S4 File Data files. (XLSX)

S1 Table Clinical scoring scheme. (DOCX)

S2 Table Histopathology scoring system. (DOCX)

S3 Table Mass spectroscopy results. Complete results of lipids measured in lymph nodes.
(XLSX)

S4 Table Mass spectroscopy results. Complete results of lipids measured in plasma.
(XLSX)

S5 Table Summary data for lipid mediators by pathway. (DOCX)

S6 Table Endocannabinoids and lipid mediator abbreviations. (DOCX)
