# Supplementary material for: A Randomized Placebo Controlled Trial of Ibuprofen for Respiratory Syncytial Virus Infection in a Bovine Model
Source: PLoS One. 2016 Apr 13;11(4):e0152913. doi: 10.1371/journal.pone.0152913 (PMC4830518; doi:10.1371/journal.pone.0152913)
Supplement: S1 Table — (DOCX) [file pone.0152913.s005.docx]

**Supporting information S1**

Clinical scoring system (from *Collie et al* with temperature weighting decreased by 100)

( Temperature -39.5) x 100 (0 if <0)

Respiratory rate x 1

Cough absent 0 present 10

Induced cough absent 0 present 10

Nasal discharge 0 to 3 Left x 10

Nasal discharge 0 to 3 Right x 10

Ocular discharge 0 to 3 Left x 10

Ocular discharge 0 to 3 Right x 10

Conjunctivitis absent 0 present 10

Adenitis absent 0 present 1

Adventitial breath sounds absent 0 present 100

Dyspnea absent 0 present 100

Mouth breathing absent 0 present 100

Depression absent 0 present 100

Anorexia absent 0 present 100

Total Clinical score is calculated by summing each component

1. Collie D. Pulmonary function changes and clinical findings associated with chronic respiratory disease in calves. Br Vet J. 1992;148(1):33-40.
