# Supplementary material for: A Randomized Placebo Controlled Trial of Ibuprofen for Respiratory Syncytial Virus Infection in a Bovine Model
Source: PLoS One. 2016 Apr 13;11(4):e0152913. doi: 10.1371/journal.pone.0152913 (PMC4830518; doi:10.1371/journal.pone.0152913)
Supplement: S2 Table — (DOCX) [file pone.0152913.s006.docx]

**Supporting information S13**

**Pathohistological scoring system**

Score =

sum (pathologist score 0 to 3 for each of neutrophils, fibrin, necrosis, thrombosis, hemorrhages, pleocytosis and epithelial cell transmigration from 12 representative slides)

(Possible range 0-252, observed range 24-53)

+

sum (pathologist score 0 to 3 for each of type II pneumocytes hyperplasia, bronchiolitis obliterans, peribronchial lymph nodes, macrophage infiltration, and interstitial thickening from the same 12 representative slides)

(Possible range 0-180 observed range 8-49)

+

2 X Percentage total lung consolidation

(Possible range 0 to 200 observed range 5 -100)

This can optionally be converted to a 0-100 scale by dividing by 632.
