# Supplementary material for: A Randomized Placebo Controlled Trial of Ibuprofen for Respiratory Syncytial Virus Infection in a Bovine Model
Source: PLoS One. 2016 Apr 13;11(4):e0152913. doi: 10.1371/journal.pone.0152913 (PMC4830518; doi:10.1371/journal.pone.0152913)
Supplement: S5 Table — (DOCX) [file pone.0152913.s009.docx]

**Lymph Tissue Peroxidation-Associated Metabolite Concentrations (pmol/gr tissue)**

| **Tissue** | **Bronchial Lymph** |  |  |  |  | **Mediastinal Lymph** |  |  |  |
| --- | --- | --- | --- | --- | --- | --- | --- | --- | --- |
| **Treatment** | **Ibuprofen (n=5)** | **Control (n=6)** | **p** | **VIP** |  | **Ibuprofen (n=8)** | **Control (n=8)** | **p** | **VIP** |
| ***COX Metabolism*** |  |  |  |  |  |  |  |  |  |
| 6-keto PGF1a | 1.72 (1.145 - 3.52) | 26.1 (12.8 - 52.2) | ‡ | 2.9 |  | 5.27 (2.07 - 9.03) | 115 (58.6 - 183) | ‡ | 2.9 |
| TXB2 | 5.31 (1.72 - 11) | 113 (40.4 - 404) | ‡ | 2.8 |  | 14.8 (4.45 - 46.9) | 473 (232 - 688) | ‡ | 2.8 |
| PGE1 | 0.636 (0.224 - 1.6) | 23.8 (13.8 - 37.4) | ‡ | 2.9 |  | 2.57 (0.265 - 11) | 111 (65.7 - 180) | ‡ | 2.8 |
| PGE2 | 11.5 (4.71 - 35.1) | 295 (185 - 407) | ‡ | 2.9 |  | 58.4 (7.38 - 243) | 1560 (1210 - 2210) | ‡ | 2.7 |
| 15-Keto PGE2 | 0.14 (0.1175 - 0.242) | 0.259 (0.1175 - 0.454) | † | 1.9 |  | 0.198 (0.118 - 0.686) | 2.39 (1.68 - 4.26) | ‡ | 2.7 |
| PGD2 | 0.573 (0.5725 - 0.5725) | 34 (20.2 - 63.3) | ‡ | 2.7 |  | 2.62 (0.81 - 43.3) | 258 (146 - 361) | ‡ | 2.8 |
| 15-deoxy PGJ2 | 0.264 (0.264 - 0.264) | 0.568 (0.264 - 0.748) | --- | 2.6 |  | 0.293 (0.23 - 0.799) | 4.86 (1.27 - 8.85) | ‡ | 2.8 |
| PGF2a - Select | 0.573 (0.5725 - 0.5725) | 13.3 (9.71 - 22.5) | ‡ | 2.8 |  | 2.81 (1.325 - 8.54) | 75.4 (47.3 - 93.5) | ‡ | 2.9 |
| 11-HETE | 11.1 (4.84 - 20.2) | 24.9 (14.7 - 43.1) | ‡ | 2.2 |  | 20.2 (8.04 - 41.9) | 86.2 (69.5 - 109) | ‡ | 2.8 |
| 9-HODE | 131 (73 - 185) | 196 (95.8 - 249) | ‡ | 2.0 |  | 133 (79.3 - 219) | 589 (311 - 1000) | ‡ | 2.7 |
| 9-HOTE | 5.93 (1.39 - 40.3) | 2.82 (1.19 - 5.7) | --- | 1.2 |  | 1.98 (1.26 - 3.09) | 1.98 (1.21 - 3.17) | --- | 0.2 |
| 9-KODE | 312 (139 - 924) | 195 (112.8 - 343) | + | 1.3 |  | --- | --- | --- | --- |
| ***12/15-LOX Metabolism*** |  |  |  |  |  |  |  |  |  |
| 15-HETE | 16.1 (5.57 - 29.8) | 21.9 (12.8 - 45.7) | --- | 1.0 |  | 26.9 (9.06 - 53.6) | 63.3 (36.8 - 92.7) | ‡ | 2.2 |
| 12-HETE | 24 (8.27 - 66.4) | 49.7 (5.65 - 408) | --- | 1.1 |  | 45.5 (35.1 - 69.4) | 237 (31.4 - 2240) | † | 1.9 |
| 12-HEPE | 0.539 (0.328 - 0.841) | 1.33 (0.338 - 6.13) | † | 1.7 |  | 0.858 (0.229 - 1.7) | 4.34 (0.515 - 29.4) | † | 1.8 |
| 13-HODE | 175 (90.9 - 251) | 173 (85.6 - 238) | --- | 0.4 |  | 176 (95.1 - 300) | 317 (162 - 541) | † | 1.8 |
| 13-HOTE | 25.5 (7.3 - 132) | 11 (4.28 - 17.9) | † | 1.5 |  | 8.58 (2.84 - 17.9) | 9.51 (4.42 - 30) | --- | 0.4 |
| 13-KODE | 140 (46.9 - 576.82) | 62.2 (11.9 - 194) | + | 1.4 |  | 50.6 (27.3 - 97.6) | 39.2 (23.7 - 66.7) | --- | 1.2 |
| ***5-LOX Metabolism*** |  |  |  |  |  |  |  |  |  |
| 5-HETE | 12.5 (6.72 - 20.1) | 12.2 (9.04 - 17.4) | --- | 0.7 |  | 22.9 (9.07 - 58.4) | 35 (24.8 - 60.2) | --- | 1.0 |
| 5-HEPE | 3.09 (1.36 - 6.97) | 2.46 (0.597 - 10.6) | --- | 0.4 |  | 1.23 (0.1625 - 7.26) | 1.35 (0.325 - 3.11) | --- | 0.8 |
| 5-KETE | 5.01 (2.7 - 8.59) | 5.44 (2.94 - 12.4) | --- | 0.4 |  | 7.05 (1.21 - 23.4) | 7.43 (4.96 - 12.5) | --- | 0.5 |
| ***ROS and Other LOX Products*** |  |  |  |  |  |  |  |  |  |
| 9,12,13-TriHOME | 18.5 (10.9 - 31.5) | 18.3 (10.1 - 29.7) | --- | 0.5 |  | 22.6 (11.6 - 44.2) | 23.4 (15.2 - 42.4) | --- | 0.3 |
| 9-HETE | 5.77 (1.99 - 10.4) | 4.82 (2.81 - 6.38) | --- | 1.2 |  | 8.51 (3.23 - 20.1) | 10.5 (8.14 - 15.8) | --- | 0.6 |
| 8-HETE | 6.68 (2.92 - 13) | 6.36 (4.725 - 7.46) | --- | 0.6 |  | 8.88 (3.86 - 15.2) | 10.3 (7.96 - 12.5) | --- | 0.7 |

Results are reported as Geometric Means (Ranges). Mean differences were determined by 2-tailed t-tests after transformation to normality. Differences are indicated at p<0.1 (+), p<0.05 †), p<0.01 (‡). Variable Importance in Projections (VIP) Scores are provided from the PLS-DA analysis using all clinical, cytokine, lymph mediator and plasma mediator results.

|  |
| --- |
|  |

**Lymph Tissue Epoxygenase-Associated Metabolite Concentrations (pmol/gr tissue)**

|  | **Bronchial Lymph** |  |  |  |  | **Mediastinal Lymph** |  | |  |  |
| --- | --- | --- | --- | --- | --- | --- | --- | --- | --- | --- |
| **Treatment** | **Ibuprofen (n=5)** | **Control (n=6)** | **p** | **VIP** |  | **Ibuprofen (n=8)** | **Control (n=8)** | | **p** | **VIP** |
| ***CYP Metabolism*** |  |  |  |  |  |  |  | |  |  |
| 12(13)-EpOME | 43.4 (9.13 - 264) | 20.4 (9.2 - 47.4) | --- | 1.3 |  | 16.7 (7.96 - 22.981) | 14.3 (7.78 - 40.3) | | --- | 0.1 |
| 9(10)-EpOME | 24.6 (5.59 - 128) | 15.1 (2.98 - 57) | --- | 0.9 |  | 15 (6.47 - 23.5) | 12.9 (6.63 - 37.276) | | --- | 0.3 |
| 14(15)-EpETrE | 7.6 (1.5 - 38) | 5.66 (1.1 - 24.5) | --- | 0.5 |  | 5.47 (0.749 - 13.4) | 6.49 (3.1 - 27) | | --- | 0.4 |
| 11(12)-EpETrE | 8.34 (2.03 - 40) | 5.31 (1.24 - 30.4) | --- | 0.7 |  | 5.44 (1.11 - 14.9) | 5.03 (2.57 - 14.6) | | --- | 0.6 |
| 8(9)-EpETrE | 22.1 (1.4 - 124) | 8.2 (0.669 - 41.8) | + | 1.3 |  | 6.63 (1.34 - 18.6) | 6.18 (1.29 - 24) | | --- | 0.4 |
| 20-HETE | 3.82 (0.9 - 9.31) | 6.01 (3.46 - 12.4) | --- | 1.0 |  | 6.5 (1.575 - 13.9) | 2.9 (1.575 - 11.8) | | + | 1.4 |
| ***sEH Metabolites*** |  |  |  |  |  |  |  | |  |  |
| 12,13-DiHOME | 0.147 (0.0983 - 0.202) | 0.0959 (0.0401 - 0.145) | ‡ | 1.9 |  | 0.145 (0.0832 - 0.976) | 0.0973 (0.0767 - 0.14) | | --- | 0.9 |
| 9,10-DiHOME | 3.81 (2.75 - 4.96) | 2.94 (1.23 - 4.3) | + | 1.3 |  | 3.76 (2.15 - 13.1) | 3.08 (2.43 - 3.96) | | --- | 0.9 |
| 14,15-DiHETrE | 1.23 (1.09 - 1.42) | 1.13 (0.687 - 1.62) | --- | 0.4 |  | 1.33 (0.931 - 2.15) | 1.31 (0.835 - 1.98) | | --- | 0.6 |
| 11,12-DiHETrE | 0.817 (0.697 - 0.939) | 0.925 (0.482 - 1.68) | --- | 1.0 |  | 1.01 (0.645 - 1.41) | 0.981 (0.711 - 1.19) | | --- | 0.7 |
| 8,9-DiHETrE | 0.535 (0.322 - 0.777) | 0.727 (0.436 - 1.38) | --- | 1.3 |  | 0.899 (0.246 - 1.25) | 0.957 (0.559 - 1.45) | | --- | 0.1 |
| 5,6-DiHETrE | 0.327 (0.0677 - 0.535) | 0.276 (0.0955 - 0.691) | --- | 0.6 |  | 0.642 (0.192 - 1.04) | 0.408 (0.157 - 0.859) | | + | 1.3 |
| 17,18-DiHETE | 2.92 (0.613 - 7.23) | 3.81 (0.3065 - 6.6) | --- | 1.2 |  | 1.53 (0.2695 - 3.12) | 1.75 (0.2695 - 6.36) | | --- | 0.7 |
| 19,20-DiHDoPA | 0.643 (0.444 - 0.969) | 0.578 (0.222 - 0.915) | --- | 0.5 |  | --- | --- | | --- | --- |
| Results are reported as Geometric Means (Ranges). Mean differences were determined by 2-tailed t-tests after transformation to normality. Differences are indicated at p<0.1 (+), p<0.05 †), p<0.01 (‡). Variable Importance in Projections (VIP) Scores are provided from the PLS-DA analysis using all clinical, cytokine, lymph mediator and plasma mediator results. | | | | | | | |  |  |  |
|  |  |  |  |  |  |  |  |  |  |  |

**Lymph Tissue Monoacylglycerides and N-Acylethanolamides Concentrations (pmol/gr tissue)**

| **Tissue** | **Bronchial Lymph** |  |  |  |  | **Mediastinal Lymph** |  |  |  |
| --- | --- | --- | --- | --- | --- | --- | --- | --- | --- |
| **Treatment** | **Ibuprofen (n=5)** | **Control (n=6)** | **p** | **VIP** |  | **Ibuprofen (n=8)** | **Control (n=8)** | **p** | **VIP** |
| 1-OG | 7530 (6684 - 8163) | 6360 (5287.6 - 7760) | ‡ | 2.1 |  | --- | --- | --- | --- |
| 2-OG | 9830 (8100 - 11500) | 7400 (4890 - 10400) | ‡ | 1.9 |  | 7840 (3120 - 20200) | 6400 (3430 - 10400) | --- | 0.8 |
| 1-LG | 2390 (1999.1 - 3370) | 2650 (2090 - 3430) | --- | 0.9 |  | 1670 (695 - 2780) | 1670 (846 - 2610) | --- | 0.6 |
| 2-LG | 7350 (4980 - 14000) | 8590 (5710 - 12400) | --- | 0.7 |  | 5030 (1500 - 9890) | 4770 (1770 - 9030) | --- | 0.7 |
| 1-AG | 849 (579 - 1190) | 971 (769 - 1200) | --- | 0.9 |  | 577 (75.9 - 1690) | 717 (285 - 1480) | --- | 0.5 |
| 2-AG | 2810 (1770 - 4790) | 2990 (2090 - 4000) | --- | 0.4 |  | 1850 (224 - 5150) | 2070 (776 - 4720) | --- | 0.6 |
| PEA | 115 (93.588 - 154) | 114 (75.9 - 138) | --- | 0.5 |  | 138 (88.4 - 183) | 146 (121 - 173) | --- | 0.3 |
| SEA | 99 (73.2 - 128) | 105 (65 - 139) | --- | 0.6 |  | 114 (92 - 166) | 118 (79 - 161) | --- | 0.2 |
| OEA | 41.5 (30.7 - 58.8) | 42.9 (28.2 - 66.9) | --- | 0.3 |  | 52.4 (38.2 - 71.3) | 57.9 (38.3 - 75.1) | --- | 0.7 |
| LEA | 27.2 (19.4 - 44.3) | 25.8 (20 - 34.7) | --- | 0.6 |  | 36.6 (23.1 - 51.5) | 35.3 (28.2 - 49.6) | --- | 0.4 |
| DGLEA | 2.55 (1.82 - 3.26) | 2.85 (1.88 - 3.88) | --- | 1.0 |  | 4.06 (2.48 - 6.84) | 3.72 (2.59 - 5.39) | --- | 0.7 |
| AEA | 8.53 (4.42 - 15.9) | 9.35 (7.23 - 12) | --- | 0.6 |  | 14.7 (6.24 - 20.8) | 14.4 (10.2 - 18.7) | --- | 0.7 |
| DEA | 4.1 (3.29 - 6.4) | 4.69 (3.11 - 6.84) | --- | 0.9 |  | 6.88 (3.49 - 10.4) | 7.01 (5.98 - 9.71) | --- | 0.6 |
| DHEA | 1.61 (1.12 - 2.19) | 1.64 (1.49 - 1.94) | --- | 0.5 |  | 1.78 (1.16 - 2.71) | 1.63 (1.14 - 2.03) | --- | 0.6 |
| Results are reported as Geometric Means (Ranges). Mean differences were determined by 2-tailed t-tests after transformation to normality. Differences are indicated at p<0.1 (+), p<0.05 †), p<0.01 (‡). Variable Importance in Projections (VIP) Scores are provided from the PLS-DA analysis using all clinical, cytokine, lymph mediator and plasma mediator results. | | | | | | | | | |
|  |  |  |  |  |  |  |  |  |  |
|  |  |  |  |  |  |  |  |  |  |

**Plasma Monoacylglycerols and Acylethanolamides, and Acylglycine Concentrations (nM)**

|  | **Day 0** |  | **Day 3** |  | **Day 7** |  | **Day 10** |  |
| --- | --- | --- | --- | --- | --- | --- | --- | --- |
| ***Control*** | | | | | | | | |
| 1-OG | 291 (78.4 - 934) |  | 1200 (558 - 1590) |  | 667 (242 - 1600) |  | 680 (173 - 2760) |  |
| 2-OG | 47.9 (15 - 159) |  | 189 (94.8 - 264) |  | 107 (36.1 - 274) |  | 116 (29.1 - 373) |  |
| 1-LG | 413 (93.5 - 1090) |  | 1270 (805 - 1910) |  | 745 (419 - 1380) |  | 427 (123 - 1590) |  |
| 2-LG | 101 (20.6 - 279) |  | 308 (194 - 462) |  | 176 (103 - 322) |  | 110 (34.2 - 362) |  |
| 1-AG | 69.4 (36 - 119) |  | 106 (73.8 - 160) |  | 72.7 (42.3 - 111) |  | 89.7 (52.6 - 138) |  |
| 2-AG | 10.7 (5.62 - 18.9) |  | 16.8 (11.3 - 25.9) |  | 11 (6.08 - 17.6) |  | 13.1 (7.53 - 23) |  |
| OEA | 53.4 (40.8 - 65.4) |  | 52.8 (43.5 - 72) |  | 47 (26.7 - 69.9) |  | 37.1 (20.8 - 56.9) |  |
| LEA | 2.91 (2.49 - 3.48) |  | 3.48 (2.47 - 4.63) |  | 3.24 (2.14 - 4.65) |  | 3.21 (2.44 - 4.28) |  |
| AEA | 0.391 (0.341 - 0.432) |  | 0.398 (0.321 - 0.466) |  | 0.452 (0.26 - 0.701) |  | 0.487 (0.433 - 0.579) |  |
| DEA | 0.298 (0.223 - 0.418) |  | 0.347 (0.307 - 0.42) |  | 0.388 (0.327 - 0.5) |  | 0.346 (0.239 - 0.428) |  |
| NO-Gly | 11.5 (9.58 - 15.9) |  | 15.2 (10.3 - 21) |  | 12.5 (4.96 - 20.2) |  | 11.4 (6.7 - 19.1) |  |
| NA-Gly | 0.563 (0.359 - 0.82) |  | 0.825 (0.413 - 1.54) |  | 0.804 (0.5 - 1.91) |  | 0.688 (0.327 - 2.2) |  |
| ***Ibuprofen*** | | | | | | | | |
| 1-OG | 575 (204 - 2610) | --- | 647 (325 - 1120) | ‡ | 688 (411 - 1490) | --- | 643 (360 - 1730) | --- |
| 2-OG | 89.2 (32.8 - 394) | --- | 99 (39.8 - 187) | ‡ | 111 (73.1 - 215) | --- | 109 (55.7 - 309) | --- |
| 1-LG | 716 (184 - 2770) | --- | 758 (406 - 1380) | † | 786 (528 - 1240) | --- | 427 (178 - 1060) | --- |
| 2-LG | 178 (43.7 - 760) | --- | 184 (93.1 - 368) | † | 197 (146 - 301) | --- | 105 (46 - 267) | --- |
| 1-AG | 119 (93.1 - 234) | † | 87.9 (59.3 - 187) | --- | 67.5 (35.4 - 145) | --- | 70.4 (33.6 - 174) | --- |
| 2-AG | 17.5 (13.7 - 35.7) | + | 12.7 (7.46 - 27.7) | --- | 10.9 (4.87 - 18.5) | --- | 10.3 (5.4 - 22.2) | --- |
| OEA | 52 (32.4 - 67) | --- | 56.2 (39 - 79) | --- | 61.5 (39.4 - 121) | --- | 34.5 (24.2 - 68.7) | --- |
| LEA | 2.87 (2.15 - 3.85) | --- | 3.71 (2.83 - 4.36) | --- | 4.02 (2.74 - 6.58) | --- | 3.26 (2.16 - 6.63) | --- |
| AEA | 0.391 (0.326 - 0.511) | --- | 0.391 (0.323 - 0.523) | --- | 0.54 (0.293 - 0.849) | --- | 0.428 (0.308 - 0.551) | --- |
| DEA | 0.281 (0.213 - 0.345) | --- | 0.333 (0.215 - 0.464) | --- | 0.386 (0.277 - 0.594) | --- | 0.327 (0.267 - 0.535) | --- |
| NO-Gly | 10.6 (8.6 - 16.8) | --- | 15.5 (8.07 - 22.4) | --- | 15.7 (8.74 - 24.4) | --- | 11.4 (6.86 - 22.8) | --- |
| NA-Gly | 0.581 (0.397 - 1.12) | --- | 0.962 (0.454 - 3.71) | --- | 0.907 (0.364 - 1.42) | --- | 0.569 (0.202 - 1.32) | --- |

Results are reported as Geometric Means (Ranges). Treatment differences in means were determined by 2-tailed t-tests after transformation to normality. Differences are indicated at p<0.1 (+), p<0.05 †), p<0.01 (‡).

**Plasma CYP Epoxygenase-Dependent Metabolite Concentrations (nM)**

|  | **Day 0** |  | **Day 3** |  | **Day 7** |  | **Day 10** |  |
| --- | --- | --- | --- | --- | --- | --- | --- | --- |
|  | ***Control*** | | | | | | |  |
| 12(13)-EpOME | 4.61 (2.14 - 14.2) |  | 9.99 (7.84 - 15.1) |  | 3.04 (1.7 - 5.91) |  | 3.11 (2.21 - 4.33) |  |
| 9(10)-EpOME | 3 (1.27 - 6.75) |  | 5.7 (3.82 - 8.14) |  | 2.31 (1.03 - 4.99) |  | 2.32 (1.65 - 3.93) |  |
| 15(16)-EpODE | 4.54 (1.7 - 22.4) |  | 12.2 (7.17 - 21.9) |  | 2.28 (1.36 - 4.44) |  | 2.01 (1.21 - 3.7) |  |
| 9(10)-EpODE | 0.588 (0.306 - 1.83) |  | 1.26 (0.654 - 2.44) |  | 0.406 (0.293 - 0.851) |  | 0.425 (0.240 - 0.746) |  |
| 14(15)-EpETrE | 0.0833 (0.0169 - 0.178) |  | 0.132 (0.0605 - 0.302) |  | 0.0916 (0.0205 - 0.255) |  | 0.117 (0.0447 - 0.224) |  |
| 11(12)-EpETrE | 0.116 (0.0712 - 0.276) |  | 0.139 (0.0569 - 0.246) |  | 0.128 (0.0396 - 0.325) |  | 0.11 (0.0581 - 0.165) |  |
|  | ***Ibuprofen*** | | | | | | |  |
| 12(13)-EpOME | 5.86 (3.27 - 11.9) | --- | 6.91 (3.36 - 14) | + | 3.93 (2.56 - 5.81) | --- | 3.72 (1.86 - 7.47) | --- |
| 9(10)-EpOME | 3.63 (1.95 - 6.29) | --- | 4.34 (2.49 - 8.37) | --- | 2.94 (1.55 - 4.51) | --- | 2.69 (1.47 - 5.01) | --- |
| 15(16)-EpODE | 6.2 (2.48 - 18.5) | --- | 6.94 (2.6 - 17) | + | 2.66 (1.36 - 4.11) | --- | 2.22 (1.45 - 5.09) | --- |
| 9(10)-EpODE | 0.642 (0.339 - 1.35) | --- | 0.91 (0.436 - 2.01) | --- | 0.496 (0.2 - 1.48) | --- | 0.527 (0.211 - 1.05) | --- |
| 14(15)-EpETrE | 0.162 (0.0502 - 0.311) | † | 0.158 (0.0936 - 0.263) | --- | 0.119 (0.0151 - 0.312) | --- | 0.0762 (0.0281 - 0.238) | --- |
| 11(12)-EpETrE | 0.118 (0.0582 - 0.2) | --- | 0.17 (0.0872 - 0.368) | --- | 0.135 (0.0661 - 0.212) | --- | 0.111 (0.0444 - 0.245) | --- |

Results are reported as Geometric Means (Ranges) of n=8/group. Treatment differences in means were determined by 2-tailed t-tests after transformation to normality. Differences are indicated at p<0.1 (+), p<0.05 †), p<0.01 (‡).

**Plasma sEH-Dependent Metabolism Metabolite Concentrations (nM)**

|  | **Day 0** |  | **Day 3** |  | **Day 7** |  | **Day 10** |  |
| --- | --- | --- | --- | --- | --- | --- | --- | --- |
|  | **Control** | | | | | | |  |
| 12,13-DiHOME | 0.137 (0.0855 - 0.26) |  | 0.157 (0.118 - 0.24) |  | 0.0588 (0.0381 - 0.102) |  | 0.0464 (0.0319 - 0.0947) |  |
| 9,10-DiHOME | 4.78 (3.11 - 9.44) |  | 5.85 (4.27 - 8.78) |  | 2.3 (1.65 - 4.72) |  | 2.08 (1.32 - 3.36) |  |
| 15,16-DiHODE | 12 (8.2 - 19.4) |  | 13.8 (9.89 - 23.2) |  | 6.37 (3.93 - 9.34) |  | 4.51 (2.19 - 11.1) |  |
| 9,10-DiHODE | 0.519 (0.258 - 1.31) |  | 0.815 (0.595 - 1.39) |  | 0.195 (0.111 - 0.475) |  | 0.158 (0.0761 - 0.386) |  |
| 14,15-DiHETrE | 0.402 (0.311 - 0.507) |  | 0.404 (0.292 - 0.596) |  | 0.465 (0.315 - 0.682) |  | 0.408 (0.287 - 0.563) |  |
| 11,12-DiHETrE | 0.483 (0.328 - 0.718) |  | 0.504 (0.335 - 0.814) |  | 0.543 (0.304 - 0.89) |  | 0.449 (0.273 - 0.671) |  |
| 8,9-DiHETrE | 0.202 (0.148 - 0.269) |  | 0.213 (0.143 - 0.297) |  | 0.211 (0.164 - 0.332) |  | 0.203 (0.136 - 0.419) |  |
| 5,6-DiHETrE | 0.252 (0.157 - 0.374) |  | 0.239 (0.107 - 0.356) |  | 0.33 (0.206 - 0.462) |  | 0.26 (0.191 - 0.341) |  |
| 17,18-DiHETE | 8.59 (5.85 - 10.7) |  | 9.03 (7.1 - 11.9) |  | 8.67 (5.85 - 11.8) |  | 8.06 (6.46 - 10.6) |  |
| 19,20-DiHDoPE | 0.849 (0.391 - 1.33) |  | 0.784 (0.329 - 1.31) |  | 0.86 (0.403 - 1.4) |  | 0.707 (0.395 - 1.07) |  |
| 9,12,13-TriHOME | 8.19 (4.55 - 13.1) |  | 13.4 (6.35 - 45.5) |  | 6.48 (4.41 - 9.32) |  | 4.97 (3.17 - 8.03) |  |
|  | **Ibuprofen** | | | | | | | |
| 12,13-DiHOME | 0.12 (0.0848 - 0.224) | --- | 0.119 (0.0895 - 0.187) | + | 0.0731 (0.0267 - 0.126) | --- | 0.0508 (0.0347 - 0.0761) | --- |
| 9,10-DiHOME | 4.45 (3.03 - 8.77) | --- | 4.94 (3.78 - 7.76) | --- | 2.86 (1.28 - 6.09) | --- | 2.15 (1.82 - 3.02) | --- |
| 15,16-DiHODE | 10 (7.21 - 15.7) | --- | 11.3 (9.37 - 16.3) | + | 6.43 (3.24 - 14.7) | --- | 4.37 (3.36 - 5.54) | --- |
| 9,10-DiHODE | 0.527 (0.356 - 0.986) | --- | 0.745 (0.526 - 1.27) | --- | 0.266 (0.109 - 0.946) | --- | 0.194 (0.0633 - 0.296) | --- |
| 14,15-DiHETrE | 0.362 (0.284 - 0.469) | --- | 0.375 (0.331 - 0.497) | --- | 0.426 (0.28 - 0.604) | --- | 0.341 (0.285 - 0.392) | + |
| 11,12-DiHETrE | 0.436 (0.348 - 0.679) | --- | 0.412 (0.335 - 0.541) | --- | 0.436 (0.197 - 0.643) | --- | 0.292 (0.207 - 0.398) | ‡ |
| 8,9-DiHETrE | 0.168 (0.117 - 0.232) | --- | 0.179 (0.145 - 0.24) | + | 0.207 (0.129 - 0.397) | --- | 0.172 (0.123 - 0.274) | --- |
| 5,6-DiHETrE | 0.274 (0.162 - 0.528) | --- | 0.238 (0.126 - 0.396) | --- | 0.364 (0.254 - 0.605) | --- | 0.243 (0.128 - 0.348) | --- |
| 17,18-DiHETE | 6.48 (4.03 - 9.39) | † | 7.52 (6.14 - 9.95) | † | 8.11 (6.94 - 11.4) | --- | 6.93 (6.23 - 7.58) | + |
| 19,20-DiHDoPE | 0.578 (0.36 - 0.901) | † | 0.63 (0.42 - 0.869) | --- | 0.769 (0.356 - 1.6) | --- | 0.498 (0.346 - 0.786) | † |
| 9,12,13-TriHOME | 7.89 (5.75 - 10.7) | --- | 10.2 (5.29 - 26.7) | --- | 6.87 (4.97 - 12) | --- | 5.18 (2.54 - 10.2) | --- |

Results are reported as Geometric Means (Ranges) of n=8/group. Treatment differences in means were determined by 2-tailed t-tests after transformation to normality. Differences are indicated at p<0.1 (+), p<0.05 †), p<0.01 (‡).

**Plasma Lipoxygenase-Associated Metabolite Profiles Metabolite Concentrations (nM)**

|  | **Day 0** |  | **Day 3** |  | **Day 7** |  | **Day 10** |  |
| --- | --- | --- | --- | --- | --- | --- | --- | --- |
|  | ***Control*** | | | | | | |  |
| 13-HODE | 65.6 (25.1 - 135) |  | 119 (64.3 - 278) |  | 75.8 (41.1 - 238) |  | 65.5 (37.2 - 143) |  |
| 13-HOTE | 4.46 (2.29 - 7.2) |  | 8.32 (6.09 - 12.4) |  | 4.31 (2.7 - 9.28) |  | 3.42 (1.3 - 6.74) |  |
| 9-HOTE | 1.48 (0.574 - 2.98) |  | 3.83 (2.02 - 7.61) |  | 2.74 (1.43 - 8.09) |  | 1.88 (0.897 - 5.25) |  |
| 15-HETE | 5.9 (2.53 - 14.1) |  | 7.89 (4.9 - 22.9) |  | 8.06 (3.8 - 21.9) |  | 6.6 (2.97 - 17.6) |  |
| 15-HEPE | 0.182 (0.119 - 0.294) |  | 0.234 (0.092 - 0.461) |  | 0.292 (0.114 - 1) |  | 0.282 (0.0781 - 0.551) |  |
| 12-HETE | 13 (5.26 - 37.6) |  | 10.5 (4.44 - 25) |  | 9.62 (5.67 - 24) |  | 10.8 (4.42 - 18.6) |  |
| 12-HEPE | 0.374 (0.181 - 0.919) |  | 0.322 (0.191 - 0.781) |  | 0.384 (0.17 - 1.05) |  | 0.397 (0.277 - 0.582) |  |
| 8-HETE | 4.76 (1.87 - 13.5) |  | 7.34 (4.67 - 19.5) |  | 7.13 (2.98 - 21.4) |  | 4.7 (1.26 - 16.8) |  |
| 17-HDoHE | 3.65 (1.16 - 12.9) |  | 5.51 (2.58 - 9.67) |  | 5.07 (2.08 - 10.6) |  | 3.6 (1.34 - 14.1) |  |
| 8,15-DiHETE | 1.36 (0.42 - 4.09) |  | 1.49 (0.281 - 9.6) |  | 1.31 (0.758 - 3.73) |  | 1.91 (0.881 - 5.5) |  |
| 5-HETE | 215 (83.7 - 514) |  | 194 (86.9 - 527) |  | 231 (113 - 547) |  | 204 (74.8 - 400) |  |
| 5-HEPE | 4.92 (2.25 - 11) |  | 5.55 (2.7 - 12.6) |  | 7.42 (3.19 - 16.3) |  | 5.31 (1.96 - 11.9) |  |
| 5,15-DiHETE | 3.31 (1.56 - 12.8) |  | 3.37 (1.38 - 17.4) |  | 4.66 (1.87 - 17.8) |  | 4.14 (1.24 - 11.5) |  |
| 4-HDoHE | 165 (133 - 197) |  | 126 (103 - 160) |  | 126 (64 - 168) |  | 125 (62.2 - 188) |  |
|  | ***Ibuprofen*** | | | | | | | |
| 13-HODE | 82 (55 - 155) | --- | 86.8 (44 - 254) | --- | 92.2 (51.8 - 179) | --- | 89.7 (34.4 - 226) | --- |
| 13-HOTE | 5.3 (3.38 - 7.99) | --- | 5.97 (3.41 - 10.2) | + | 5.19 (3.02 - 9.53) | --- | 4.63 (2.74 - 8.75) | --- |
| 9-HOTE | 2.2 (1.26 - 3.68) | --- | 2.49 (1.13 - 6.41) | --- | 2.96 (2.02 - 5.69) | --- | 2.72 (1.05 - 7.07) | --- |
| 15-HETE | 7.84 (5.97 - 11.1) | --- | 7 (3.54 - 21.6) | --- | 8.55 (6.01 - 12.1) | --- | 8.13 (2.63 - 18.3) | --- |
| 15-HEPE | 0.182 (0.049 - 0.289) | --- | 0.19 (0.0509 - 1.03) | --- | 0.325 (0.183 - 0.553) | --- | 0.267 (0.0904 - 0.619) | --- |
| 12-HETE | 13.7 (8.07 - 21.8) | --- | 9.1 (4.91 - 22.3) | --- | 10.6 (7.28 - 16.9) | --- | 13.7 (6.56 - 32.8) | --- |
| 12-HEPE | 0.341 (0.18 - 0.629) | --- | 0.335 (0.199 - 0.756) | --- | 0.376 (0.231 - 0.724) | --- | 0.401 (0.22 - 1.06) | --- |
| 8-HETE | 6.26 (4.33 - 9.71) | --- | 5.47 (2.71 - 19.2) | --- | 6.97 (3.67 - 13.4) | --- | 6.92 (2.21 - 19.4) | --- |
| 17-HDoHE | 4.55 (2.65 - 6.92) | --- | 3.78 (1.29 - 10.6) | --- | 5.2 (3.32 - 11.2) | --- | 4.03 (1.86 - 11.5) | --- |
| 8,15-DiHETE | 1.15 (0.544 - 1.9) | --- | --- | --- | 1.27 (0.699 - 2.64) | --- | 2.15 (0.729 - 8.4) | --- |
| 5-HETE | 246 (115 - 457) | --- | 209 (87.1 - 636) | --- | 277 (141 - 463) | --- | 267 (69 - 479) | --- |
| 5-HEPE | 5.78 (3.56 - 8.05) | --- | 5.29 (1.88 - 12.2) | --- | 7.34 (3.26 - 14.5) | --- | 7 (2.03 - 12) | --- |
| 5,15-DiHETE | 3.58 (1.16 - 10.2) | --- | 3.47 (1.25 - 16) | --- | 5.53 (2.47 - 11.1) | --- | 5.32 (0.937 - 15.7) | --- |
| 4-HDoHE | 143 (72.1 - 210) | --- | 106 (21.5 - 218) | --- | 169 (137 - 252) | + | 141 (79.7 - 263) | --- |

Results are reported as Geometric Means (Ranges) of n=8/group. Treatment differences in means were determined by 2-tailed t-tests after transformation to normality. Differences are indicated at p<0.1 (+), p<0.05 †), p<0.01 (‡).

**Plasma Fatty Acid Alcohol Dehydrogenase-Associated Metabolite Profiles Metabolite Concentrations (nM)**

|  | **Day 0** |  | **Day 3** |  | **Day 7** |  | **Day 10** |  |
| --- | --- | --- | --- | --- | --- | --- | --- | --- |
|  | ***Control*** | | | | | | | |
| 13-KODE | 7.56 (2.94 - 21.9) |  | 16.3 (4.68 - 53.4) |  | 9.25 (5.22 - 29.3) |  | 6.55 (3.52 - 14.9) |  |
| 9-KODE | 12.7 (4.46 - 27.7) |  | 29.8 (12 - 90.7) |  | 15 (8.77 - 29) |  | 11.7 (6.92 - 18.8) |  |
| 12(13)-Ep-9-KODE | 4.49 (2.59 - 7.27) |  | 9.2 (2.81 - 32.3) |  | 4.27 (2.37 - 6.38) |  | 2.46 (1.06 - 5.59) |  |
| 15-KETE | 0.868 (0.169 - 1.97) |  | 1.13 (0.677 - 2.43) |  | 0.785 (0.453 - 1.73) |  | 0.933 (0.305 - 1.68) |  |
| 5-KETE | 5.03 (1.6 - 16.5) |  | 5.32 (2.52 - 22.7) |  | 4.61 (2.01 - 12.9) |  | 3.38 (1.42 - 6.09) |  |
|  | ***Ibuprofen*** | | | | | | | |
| 13-KODE | 9.82 (5.45 - 25.9) | --- | 11.3 (5.62 - 44.7) | --- | 11.1 (5.11 - 44.2) | --- | 8.62 (3.27 - 25.1) | --- |
| 9-KODE | 17.6 (9.07 - 50.1) | --- | 21.7 (11.4 - 77.7) | --- | 18.1 (11.4 - 102) | --- | 11.1 (6.58 - 25.5) | --- |
| 12(13)-Ep-9-KODE | 5.16 (2.27 - 12.2) | --- | 4.49 (2.32 - 19.8) | + | 5.36 (2.08 - 38.6) | --- | 2.77 (1.64 - 5.03) | --- |
| 15-KETE | 1.06 (0.49 - 2.01) | --- | 0.986 (0.66 - 2.87) | --- | 1.22 (0.667 - 2.94) | --- | 0.959 (0.389 - 2.43) | --- |
| 5-KETE | 7.03 (3.12 - 9.76) | --- | 6.1 (2.69 - 13.5) | --- | 7.81 (4.79 - 18.5) | --- | 6.19 (1.14 - 12.5) | † |

Results are reported as Geometric Means (Ranges) of n=8/group. Treatment differences in means were determined by 2-tailed t-tests after transformation to normality. Differences are indicated at p<0.1 (+), p<0.05 †), p<0.01 (‡).

**Plasma COX Metabolite Concentrations (nM)**

|  | **Day 0** |  | **Day 3** |  | **Day 7** |  | **Day 10** |  |
| --- | --- | --- | --- | --- | --- | --- | --- | --- |
|  | ***Control*** |  |  |  |  |  |  |  |
| ***COX Metabolism*** |  |  |  |  |  |  |  |  |
| TXB2 | 0.0512 (0.024 - 0.101) |  | 0.0785 (0.0261 - 0.317) |  | 0.0637 (0.0376 - 0.104) |  | 0.396 (0.0598 - 2.48) |  |
| PGD2 | 0.051 (0.0145 - 0.102) |  | 0.0838 (0.024 - 0.349) |  | 0.0859 (0.0448 - 0.142) |  | 0.0746 (0.0386 - 0.192) |  |
| 11-HETE | 4.58 (1.99 - 10.4) |  | 5.28 (3.15 - 14) |  | 5.88 (2.81 - 19) |  | 5.01 (1.97 - 14) |  |
| 9-HODE | 66.1 (25 - 153) |  | 132 (65.1 - 360) |  | 86.9 (45.8 - 280) |  | 72.2 (34.8 - 185) |  |
|  | ***Ibuprofen*** |  |  |  |  |  |  |  |
| TXB2 | 0.0673 (0.038 - 0.165) | --- | 0.0683 (0.0413 - 0.0981) | --- | 0.0378 (0.0223 - 0.0647) | † | 0.0986 (0.0212 - 0.0695) | + |
| PGD2 | 0.049 (0.0205 - 0.123) | --- | 0.0687 (0.0204 - 0.181) | --- | 0.0649 (0.0192 - 0.15) | --- | 0.0925 (0.0226 - 0.428) | --- |
| 11-HETE | 5.64 (4.02 - 9.58) | --- | 4.48 (2.12 - 15.9) | --- | 6.14 (3.99 - 12.1) | --- | 5.68 (1.82 - 17) | --- |
| 9-HODE | 87 (58.2 - 140) | --- | 95 (47.8 - 296) | --- | 104 (56.1 - 175) | --- | 104 (31.2 - 297) | --- |

Results are reported as Geometric Means (Ranges) of n=8/group. Treatment differences in means were determined by 2-tailed t-tests after transformation to normality. Differences are indicated at p<0.1 (+), p<0.05 †), p<0.01 (‡).

**Plasma Monoacylglycerols and Acylethanolamides, and Acylglycine Concentrations (nM)**

|  | **Day 0** |  | **Day 3** |  | **Day 7** |  | **Day 10** |  |
| --- | --- | --- | --- | --- | --- | --- | --- | --- |
|  | ***Control*** | | | | | | | |
| 1-OG | 291 (78.4 - 934) |  | 1200 (558 - 1590) |  | 667 (242 - 1600) |  | 680 (173 - 2760) |  |
| 2-OG | 47.9 (15 - 159) |  | 189 (94.8 - 264) |  | 107 (36.1 - 274) |  | 116 (29.1 - 373) |  |
| 1-LG | 413 (93.5 - 1090) |  | 1270 (805 - 1910) |  | 745 (419 - 1380) |  | 427 (123 - 1590) |  |
| 2-LG | 101 (20.6 - 279) |  | 308 (194 - 462) |  | 176 (103 - 322) |  | 110 (34.2 - 362) |  |
| 1-AG | 69.4 (36 - 119) |  | 106 (73.8 - 160) |  | 72.7 (42.3 - 111) |  | 89.7 (52.6 - 138) |  |
| 2-AG | 10.7 (5.62 - 18.9) |  | 16.8 (11.3 - 25.9) |  | 11 (6.08 - 17.6) |  | 13.1 (7.53 - 23) |  |
| OEA | 53.4 (40.8 - 65.4) |  | 52.8 (43.5 - 72) |  | 47 (26.7 - 69.9) |  | 37.1 (20.8 - 56.9) |  |
| LEA | 2.91 (2.49 - 3.48) |  | 3.48 (2.47 - 4.63) |  | 3.24 (2.14 - 4.65) |  | 3.21 (2.44 - 4.28) |  |
| AEA | 0.391 (0.341 - 0.432) |  | 0.398 (0.321 - 0.466) |  | 0.452 (0.26 - 0.701) |  | 0.487 (0.433 - 0.579) |  |
| DEA | 0.298 (0.223 - 0.418) |  | 0.347 (0.307 - 0.42) |  | 0.388 (0.327 - 0.5) |  | 0.346 (0.239 - 0.428) |  |
| NO-Gly | 11.5 (9.58 - 15.9) |  | 15.2 (10.3 - 21) |  | 12.5 (4.96 - 20.2) |  | 11.4 (6.7 - 19.1) |  |
| NA-Gly | 0.563 (0.359 - 0.82) |  | 0.825 (0.413 - 1.54) |  | 0.804 (0.5 - 1.91) |  | 0.688 (0.327 - 2.2) |  |
|  | ***Ibuprofen*** | | | | | | | |
| 1-OG | 575 (204 - 2610) | --- | 647 (325 - 1120) | ‡ | 688 (411 - 1490) | --- | 643 (360 - 1730) | --- |
| 2-OG | 89.2 (32.8 - 394) | --- | 99 (39.8 - 187) | ‡ | 111 (73.1 - 215) | --- | 109 (55.7 - 309) | --- |
| 1-LG | 716 (184 - 2770) | --- | 758 (406 - 1380) | † | 786 (528 - 1240) | --- | 427 (178 - 1060) | --- |
| 2-LG | 178 (43.7 - 760) | --- | 184 (93.1 - 368) | † | 197 (146 - 301) | --- | 105 (46 - 267) | --- |
| 1-AG | 119 (93.1 - 234) | † | 87.9 (59.3 - 187) | --- | 67.5 (35.4 - 145) | --- | 70.4 (33.6 - 174) | --- |
| 2-AG | 17.5 (13.7 - 35.7) | + | 12.7 (7.46 - 27.7) | --- | 10.9 (4.87 - 18.5) | --- | 10.3 (5.4 - 22.2) | --- |
| OEA | 52 (32.4 - 67) | --- | 56.2 (39 - 79) | --- | 61.5 (39.4 - 121) | --- | 34.5 (24.2 - 68.7) | --- |
| LEA | 2.87 (2.15 - 3.85) | --- | 3.71 (2.83 - 4.36) | --- | 4.02 (2.74 - 6.58) | --- | 3.26 (2.16 - 6.63) | --- |
| AEA | 0.391 (0.326 - 0.511) | --- | 0.391 (0.323 - 0.523) | --- | 0.54 (0.293 - 0.849) | --- | 0.428 (0.308 - 0.551) | --- |
| DEA | 0.281 (0.213 - 0.345) | --- | 0.333 (0.215 - 0.464) | --- | 0.386 (0.277 - 0.594) | --- | 0.327 (0.267 - 0.535) | --- |
| NO-Gly | 10.6 (8.6 - 16.8) | --- | 15.5 (8.07 - 22.4) | --- | 15.7 (8.74 - 24.4) | --- | 11.4 (6.86 - 22.8) | --- |
| NA-Gly | 0.581 (0.397 - 1.12) | --- | 0.962 (0.454 - 3.71) | --- | 0.907 (0.364 - 1.42) | --- | 0.569 (0.202 - 1.32) | --- |

Results are reported as Geometric Means (Ranges) of n=8/group. Treatment differences in means were determined by 2-tailed t-tests after transformation to normality. Differences are indicated at p<0.1 (+), p<0.05 †), p<0.01 (‡).
