# Supplementary material for: A Randomized Placebo Controlled Trial of Ibuprofen for Respiratory Syncytial Virus Infection in a Bovine Model
Source: PLoS One. 2016 Apr 13;11(4):e0152913. doi: 10.1371/journal.pone.0152913 (PMC4830518; doi:10.1371/journal.pone.0152913)
Supplement: S6 Table — (DOCX) [file pone.0152913.s010.docx]

| **Endocannabinoids** | |  |  |
| --- | --- | --- | --- |
| **Parent Lipid** | **Class** | **HMDB ID** | **Common Abbreviaton** |
| C16:0 | Ethanolamide | HMDB02100 | PEA |
| C18:0 | Ethanolamide | HMDB13078 | SEA |
| C18:1n9 | Ethanolamide | HMDB02088 | OEA |
| C18:2n6 | Ethanolamide | HMDB12252 | LEA |
| C18:3n3 | Ethanolamide | HMDB13624 | aLEA |
| C20:3n6 | Ethanolamide | HMDB13625 | DGLA EA |
| C20:4n6 | Ethanolamide | HMDB04080 | AEA |
| C22:4n6 | Ethanolamide | HMDB13626 | DEA |
| C22:6n3 | Ethanolamide | HMDB13627 | DHEA |
| PGF2a | Ethanolamide | HMDB13628 | PGF2a EA |
| PGD2 | Ethanolamide | HMDB13629 | PGD2 EA |
| 20-HETE | Ethanolamide | HMDB13630 | 20-HETE EA |
| C18:1n9 | 1-Acyl Glycerol | HMDB11567 | 1-OG |
| C18:2n6 | 1-Acyl Glycerol | HMDB11568 | 1-LG |
| C20:4n6 | 1-Acyl Glycerol | HMDB11578 | 1-AG |
| C18:1n9 | 2-Acyl Glycerol | HMDB11537 | 2-OG |
| C18:2n6 | 2-Acyl Glycerol | HMDB11538 | 2-LG |
| C20:4n6 | 2-Acyl Glycerol | HMDB04666 | 2-AG |
| C18:1n9 | N-Acyl Glycine | HMDB13631 | NO-Gly |
| 20:4n6 | N-Acyl Glycine | HMDB05096 | NA-Gly |

Endocannabinoid abbreviations .
